# Supplementary material for: Neuromorphic electro-stimulation based on atomically thin semiconductor for damage-free inflammation inhibition
Source: Nat Commun. 2024 Feb 13;15:1327. doi: 10.1038/s41467-024-45590-8 (PMC10864345; doi:10.1038/s41467-024-45590-8)
Supplement: Supplementary file 1 — Supplementary Information [file 41467_2024_45590_MOESM1_ESM.pdf]

Supplementary Materials

Neuromorphic Electro-Stimulation Based on Atomically Thin Semiconductor for Damage-Free Inflammation Inhibition

Rong Bao[1,†], Shuiyuan Wang[2,†,*], Xiaoxian Liu[2,†], Kejun Tu[3], Jingquan Liu[3], Xiaohe Huang[2], Chunsen Liu[2], Peng Zhou [2,*] and Shen Liu[1,*]

[1]Shanghai Sixth People's Hospital Affiliated to Shanghai Jiao Tong University School of Medicine, Shanghai 200025, China

[2]Shanghai Key Lab for Future Computing Hardware and System, School of Microelectronics, Fudan University, Shanghai 200433, China

[3]National Key Laboratory of Science and Technology on Micro/Nano Fabrication, DCI Joint Team, Collaborative Innovation Center of IFSA, Department of Micro/Nano Electronics, Shanghai Jiao Tong university, Shanghai 200240, China

†These authors contributed equally to this work

*Author to whom correspondence should be addressed: sy_wang@fudan.edu.cn, pengzhou@fudan.edu.cn, liushensjtu@sjtu.edu.cn

Table of Contents

Section 1 | Neuromorphic electrostimulator protocol……………………………......................3
Section 2 | Detailed fabrication flows……………………..3
Section 3 | Optical and SEM characterization……………...4
Section 4 | Raman and AFM characterization..………...5
Section 5 | Repeated transfer/leakage curves and endurance..7
Section 6 | Pulses with different widths………………..………………….......................8
Section 7 | Conductance switching mechanism………..9
Section 8 | Retention characteristics of 2D MoS_2 FGM…...10
Section 9 | LTP/LTD response curves…………………..………............................... 11
Section 10 | Signals from commercial stimulator and sympathetic nerve...........................12
Section 11 | Bionic stimulus spikes with gate voltage variation………..……….............13
Section 12 | Charge balancing in the neuromorphic stimulation system…………………15
Section 13 | Current climbing and real-time monitoring……………….............................14
Section 14 | Injection of PRV...17
Section 15 | Immunostaining of TH...18
Section 16 | Electrode implantation…...19
Section 17 | The influence of implantation……………………………………………….20
Section 18 | Low stimulation current caused little nerve damage......................................21
Section 19 | Comprehensive assessment of damage after ES……………………………22
Section 20 | Stimulation current threshold………………………................................25
Section 21 | IL-6$^+$ and CD68$^+$ cell concentrations………..…………………………..…....26
Section 22 | Comprehensive analysis of inflammatory cytokines………………………..27
Section 23 | Determine the effects of 2D ES on blood flow………...................................28
Section 24 | KEGG and GO analysis..29
Section 25 | ADRB2$^+$CD68$^+$ cell concentration………..30
Section 26 | The effect of antagonists against ADRB2…………………………………...31
Section 27 | Benchmarking for electrostimulator systems…………………………….......32

Section 1. Neuromorphic electrostimulator protocol

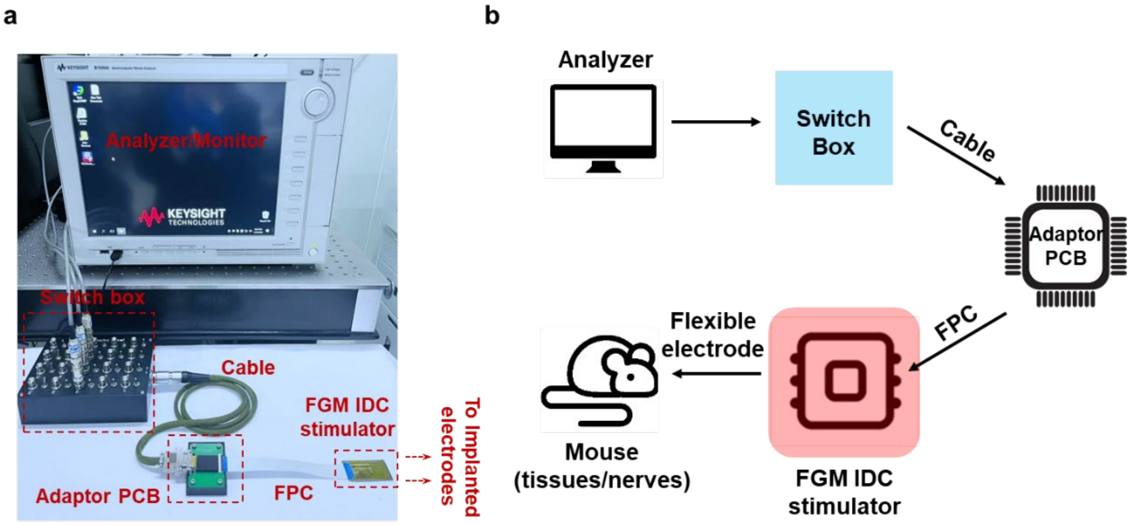

Figure S1 | Set-up and schematic of the neuromorphic electrostimulator system. The core components of the proposed neuromorphic electrostimulator system include 2D MoS_2 FGM IDC stimulator with a scalable length of implanted electrodes, the FPC, the adapter PCB, the switch box and the analyzer/monitor. **a,** Set-up of the neuromorphic electrostimulator system. **b,** Working schematic of the neuromorphic electrostimulator system.

Section 2. Detailed fabrication flows

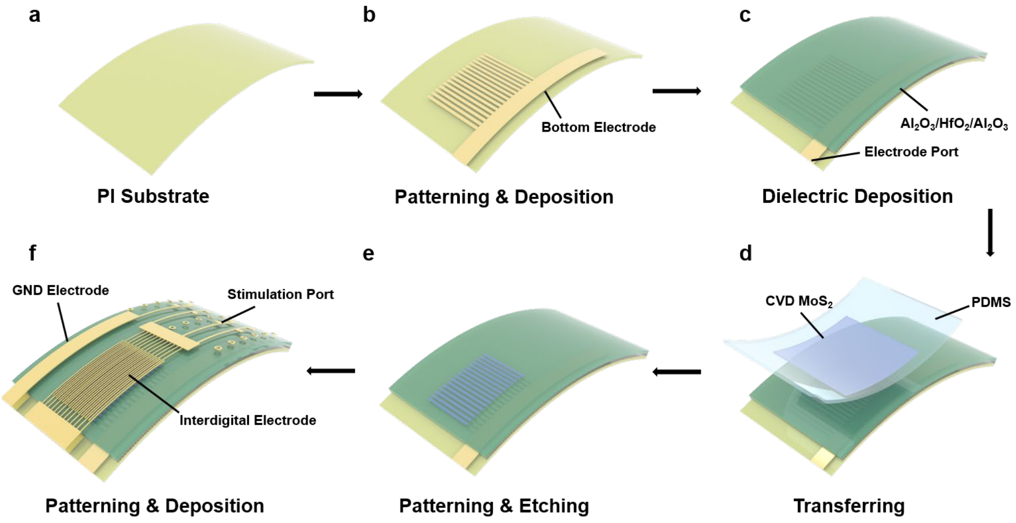

Figure S2 | Fabrication flow of the 2D FGM IDC. a, Cleaning of flexible PI substrate, thickness of PI is 30 μm. **b,** The bottom gate pattern is determined using laser direct imaging (LDI) lithography and the metal is deposited using electron beam evaporation (EBE). **c,** Formation of $Al_2O_3/HfO_2/Al_2O_3$ stacked dielectric layers using atomic layer deposition (ALD)[1]. **d,** Transfer of 2D semiconductor MoS_2 film prepared by chemical vapor deposition (CVD). **e,** Patterning and etching of MoS_2 films to form discrete channels. **f,** Define interdigital electrode patterns and deposit metal for ES.

Section 3. Optical and SEM characterization

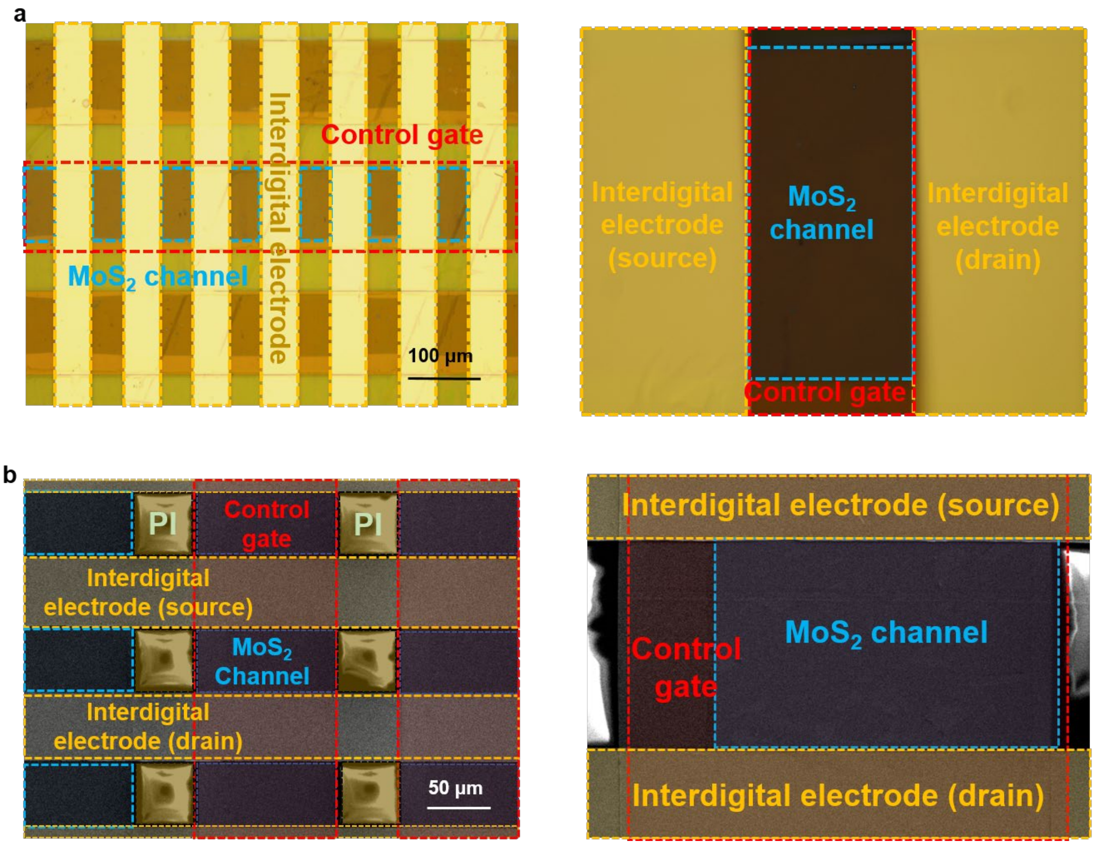

Figure S3 | Optical and SEM images of the prepared FGM IDC. a, Optical images of the 2D FGM IDC at different magnifications, scale bar: 100 μm. **b,** SEM images of the 2D FGM IDC at different magnifications, scale bar: 50 μm. The images on the right are enlargements of individual interdigital channel, the blue, red and yellow dashed boxes represent the MoS_2 channel, control gate and interdigital electrodes, respectively. The optical and SEM were repeated three times with similar results.

Section 4. Raman and AFM characterization

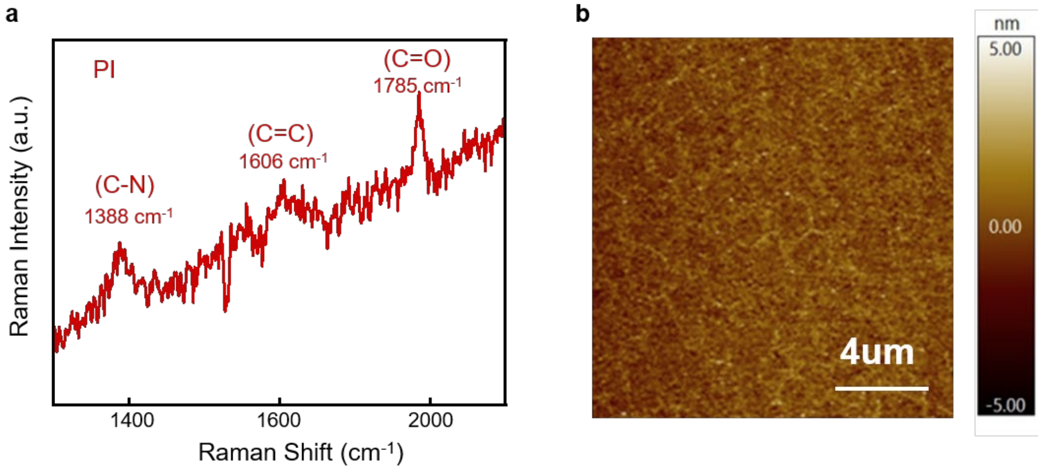

Figure S4 | Raman spectra of PI substrate and AFM characterization of 2D FGM channel. a, PI shows three distinct peaks at 1388, 1606, and 1785 cm^{-1}, corresponding to the C-N, C=C, and C=O bonds, respectively. The wavelength of excitation laser is 532 nm. **b,** The AFM image shows a flat and uniform surface of monolayer MoS_2 channel, scale bar: 4 μm. The AFM was repeated three times with similar results.

Section 5. Repeated transfer/leakage curves and endurance

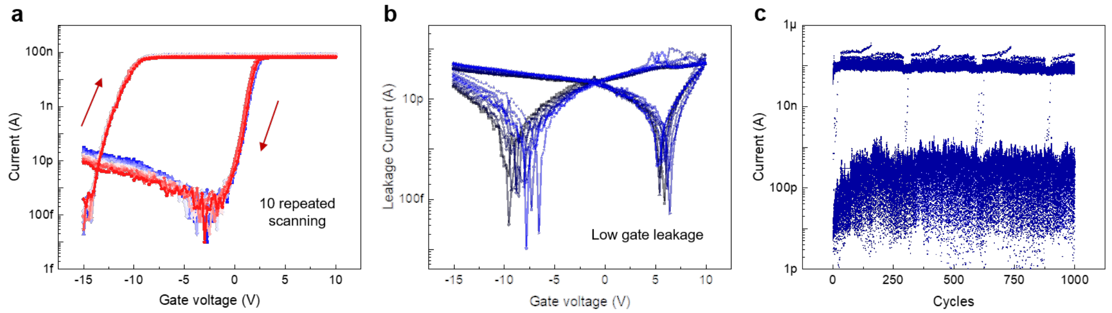

Figure S5 | The transfer/leakage and endurance characteristics of 2D FGM. a, Repeated 10-cycle transfer curves for single-channel stimulated devices, which show stable thresholds and large hysteresis windows. The gate voltage sweep is from -15 V to +10 V, with drain voltage fixed at 1 V. **b,** The leakage characteristics corresponding to the 10 repeated scanning. The results show that the gate exhibits a low leakage current in the range of 100 fA~10 pA. **c,** The conductance exhibits reliable LRS and HRS switching within 1000 operating cycles, which indicates good durability of 2D FGM. Programming/erasing amplitude for endurance testing is ±10 V, width is 10 ms, and reading duration is 1 s. Drain voltage is fixed at 1 V.

Section 6. Pulses with different widths

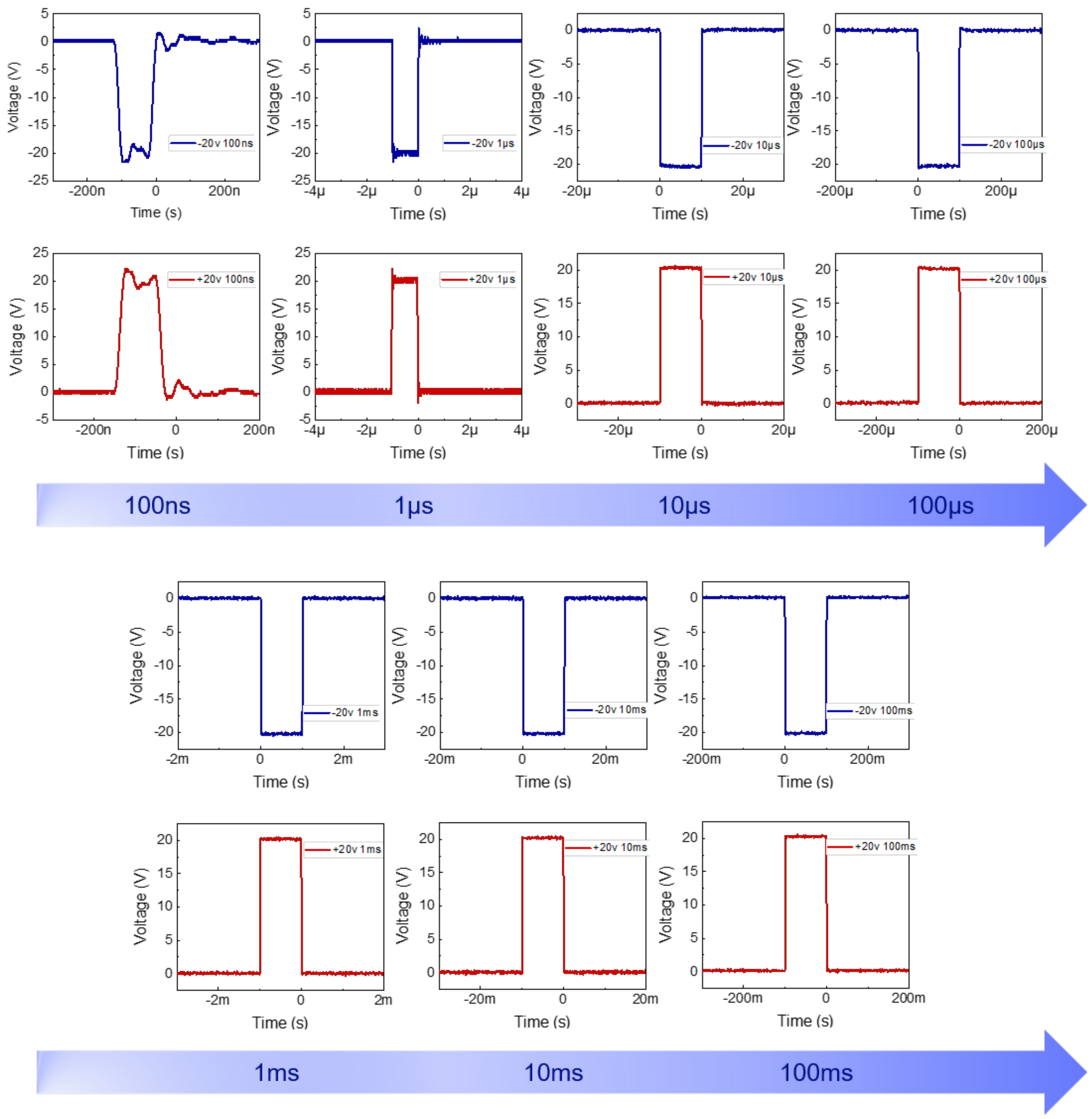

Figure S6 | Application of pulses with different widths. Fig. S6 shows pulses with different pulse widths from 100 ns to 100 ms, which corresponds to the actual pulses applied in the operating speed of **Fig. 3b** in the main text.

Section 7. Conductance switching mechanism

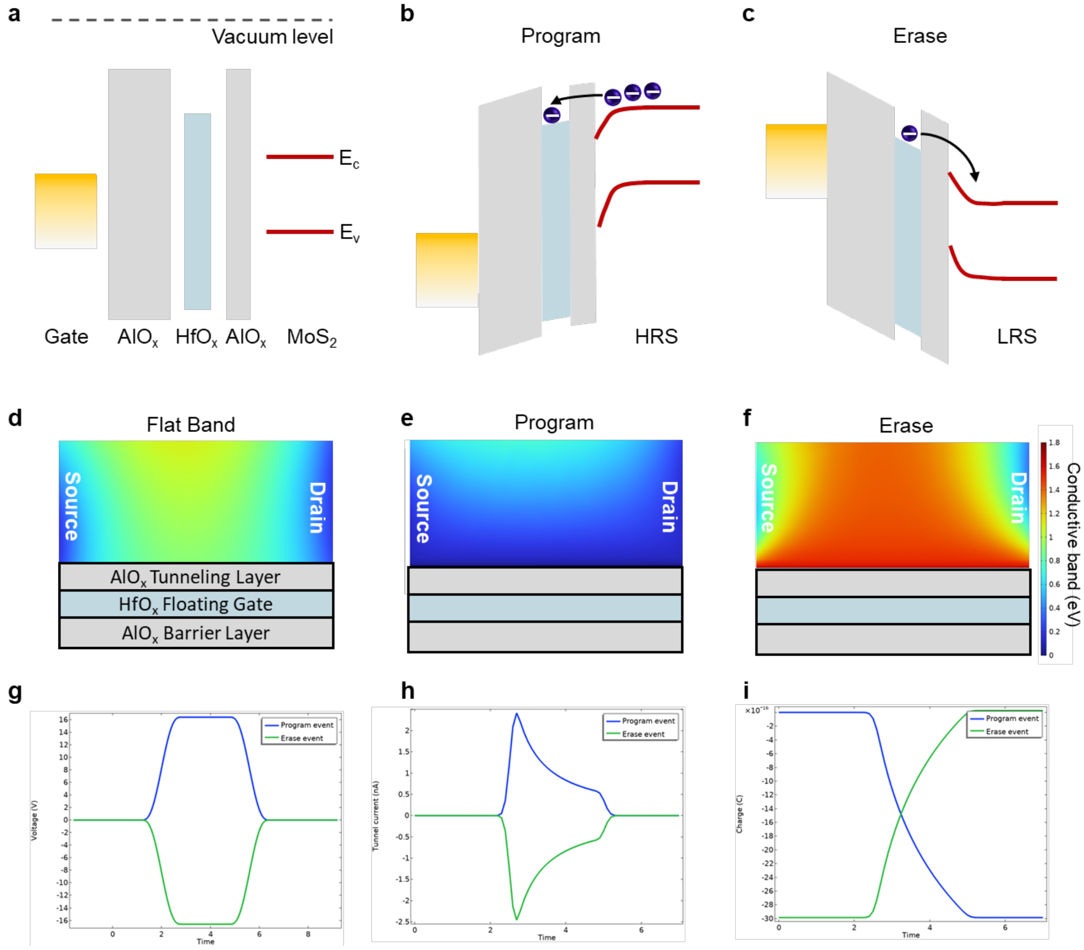

Figure S7 | Energy band of 2D FGM. **a,** Energy bands of the different materials composing the 2D FGM prior to contact[2]. E_c and E_v are the positions of the bottom of the MoS_2 conduction band and the top of the valence band, respectively. Stacked $AlO_x/HfO_x/AlO_x$ sequentially acts as a blocking, trapping and tunneling layer. **b,** The 2D FGM is programmed by applying a positive gate voltage to inject electrons into the HfO_x (floating gate), thus enabling the HRS. **c**, Erase operation to extract electrons from the floating gate with negative gate voltage applied, thus switching back to LRS. **d**, The simulated energy band of 2D FGM in flat band state. **e**, The simulated energy band of 2D FGM in program state. A downward bend energy band could be observed. **f**, The simulated energy band of 2D FGM in erase state. **g**, The voltage pulse used in program and erase event. **h**, The tunneling current in program and erase event, which indicates the electron's moving through barrier layer. **i**, The trapped charge in the floating gate.

Section 8. Retention characteristics of 2D MoS_2 FGM

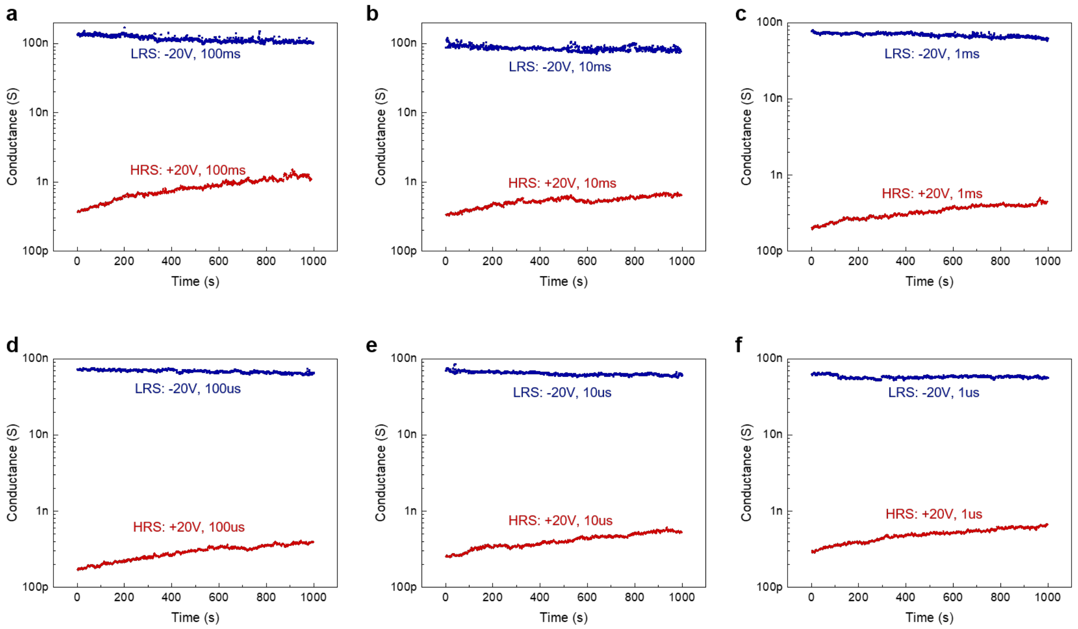

Figure S8 | Retention characteristics at different pulse widths. a~f, The retention characteristics under 1 μs~100 ms pulses, respectively. The programming/erasing amplitude is fixed at ±20 V.

Section 9. LTP/LTD response curves

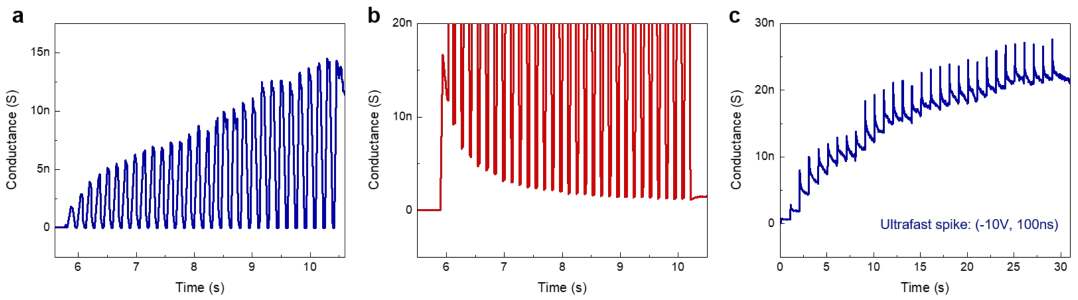

Figure S9 | Original response curves of LTP/LTD under 30 stimulus spikes. a, LTP response of the 2D electrostimulator device under spike stimulation with an amplitude of -6 V. **b,** LTD response of the 2D electrostimulator device under spike stimulation with an amplitude of +1 V. **c,** LTP response to an ultrafast spike stimulus with an amplitude of -10 V and a width of 100 ns. Drain voltage is fixed at 1 V.

Section 10. Signals from commercial stimulator and sympathetic nerve

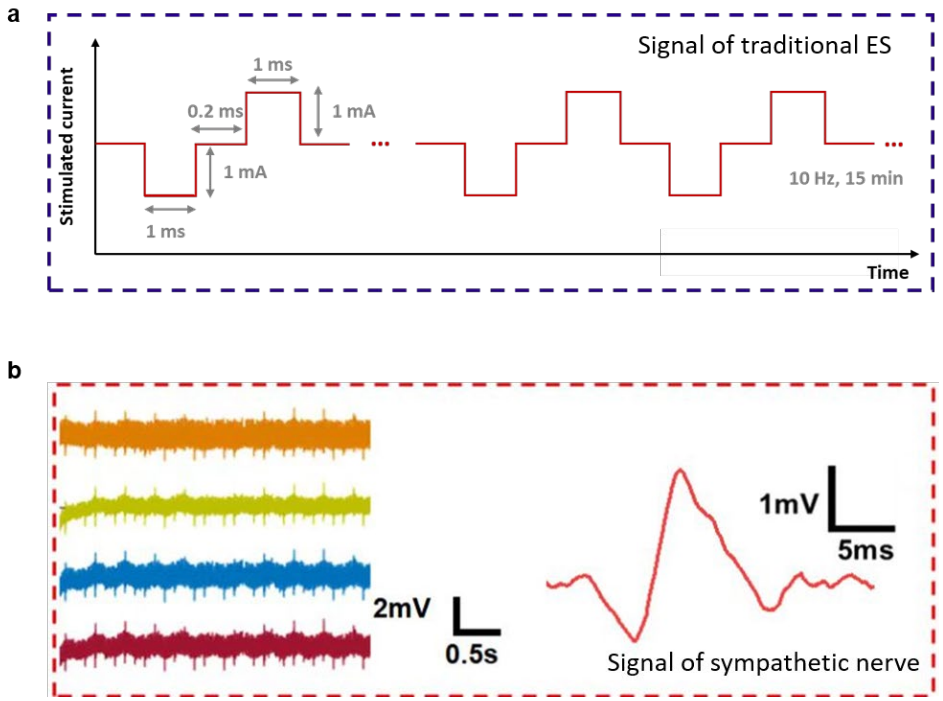

Figure S10 | Commercial stimulator waveforms and sympathetic nerve biosignals. a, Typical fixed rectangular charged-balanced biphasic current pulses stimulation provided by commercial stimulators. Stimulation amplitude is 1 mA, pulse width is 1 ms, frequency is 10 Hz, duration is 15 min. **b,** Biological signals of sympathetic nerves.

Section 11. Bionic stimulus spikes with gate voltage variation

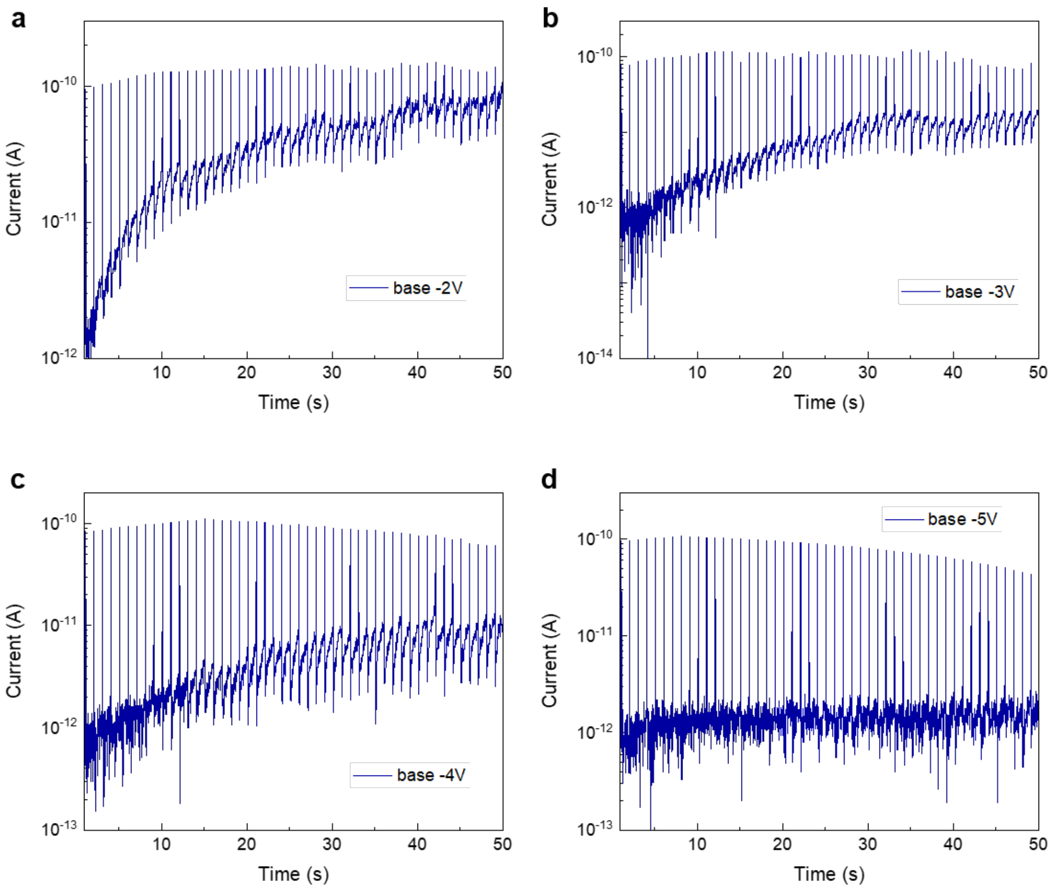

Figure S11 | bionic stimulus spikes under gate base voltage variation. a~d, The bionic stimulus currents generated at gate base voltages of -2, -3, -4, and -5 V, respectively, with pulse amplitude and width fixed at +15 V for 100 ns, pulse number of 50 and drain voltage of 1 V. This shows that the bionic spikes emitted by the neuromorphic electrostimulator can be modulated by the gate base voltage.

Section 12. Charge balancing in neuromorphic stimulation system

The proposed neuromorphic ES exhibit a different charge balancing approach than the existing ES due to different working principles, as shown in **Figure S12a**. For existing stimulators, the ES waveform typically consists of a positive pulse and a negative pulse, with the positive and negative pulse currents being integrated equally in time (shaded portion of the figure), i.e., charge balance is achieved, as shown in **Figure S12b**. This biphasic waveform acts directly on biological tissues/nerves (top panel of **Figure S12a**), and the charge balance reduces the tissue damage caused by electrolysis. While For neuromorphic ES, these typical biphasic charge-balance waveforms were applied via the analyzer to the 2D FGM IDC, whose output of bionic spikes was used as a nerve stimulus (**Figure S12a** bottom panel). The bionic spikes output from the 2D FGM IDC can be programmed for charge balancing. **Figure S12c** shows representative bionic spikes generated by the neuromorphic device (extracted from **Fig. 3f**). The dashed line can be considered as the baseline of the bioelectric current induced by intracellular ions. By integrating the relative positive and negative currents, it can be calculated that the relative positive and negative charge varied in one period is ~2.4×10^{-12} C, which means that charge equilibrium has been reached. Furthermore, for neuromorphic ES, once the charge balance at the input terminals is broken (due to changes in voltage pulses), the electrolysis process and charge accumulation will take place in the FGM IDC device rather than in the biological tissues/nerves, which can serve as an additional protection.

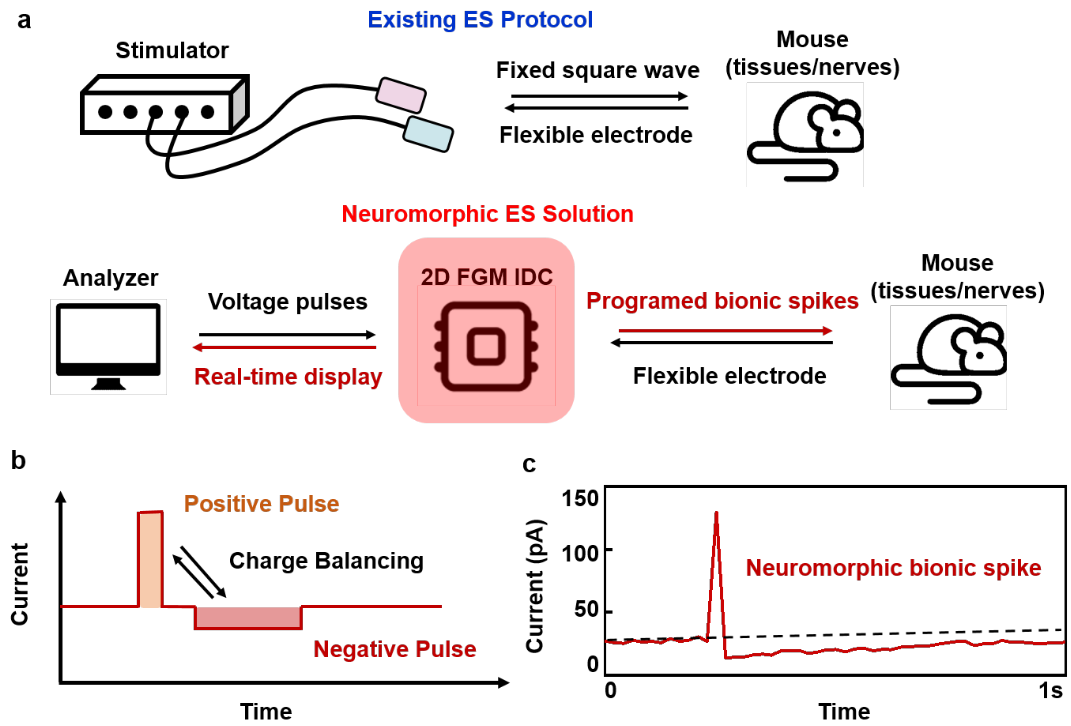

Figure S12. Charge balancing in functional electrical stimulation systems. a, Comparison of existing and neuromorphic ES solutions. For the existing ES, mouse tissues or nerves are connected to the stimulator via flexible electrodes, and the stimulator gives a charge-balanced biphasic square wave signal with a fixed stimulation amplitude; for the proposed neuromorphic ES, the mouse is connected to the 2D FGM IDC via flexible electrodes, and the analyzer applies voltage pulses to the FGM IDC to enable it to output programed bionic spikes as stimuli, while the analyzer monitors and displays the current of the stimulation loop in real time. **b,** Typical waveform of an existing stimulator. The waveform contains positive and negative current pulses that can be tuned in duration and amplitude to achieve charge balance. **c**, Representative bionic spike waveform in neuromorphic ES, which can be programmed for charge balancing. The bionic spike was repeated three times with similar results.

Section 13. Current climbing and real-time monitoring

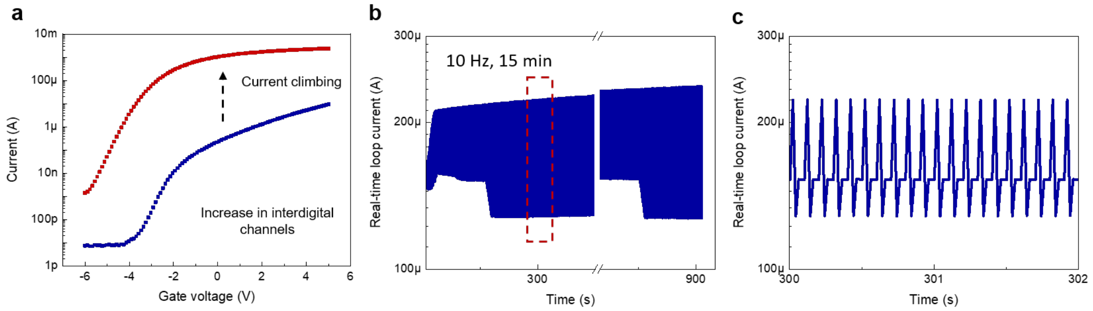

Figure S13 | Interdigital current climbing effect and real-time monitoring of stimulus loop. a, The output current of 2D FGM can increase up to the mA level with the accumulation of interdigital channels. **b,** Real-time current for 10 Hz 15 min stimulation. **c,** Enlarged view of the loop current at the position of red dashed box.

Section 14. Injection of PRV

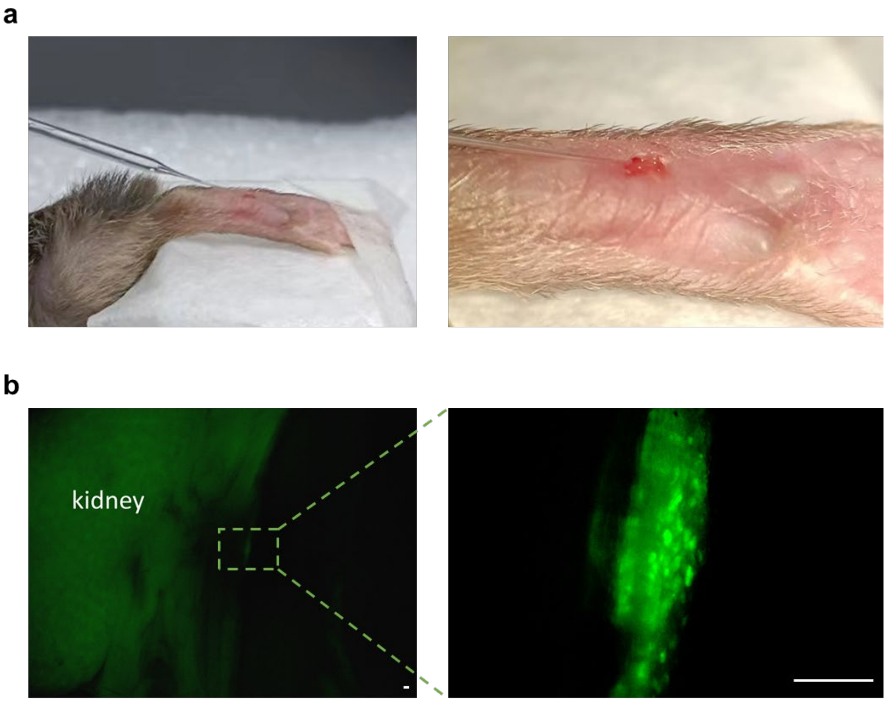

Figure S14 | Injection of PRV. a, The schematic of injection. A tiny cut was made under hind paw and 3μl PRV-EGFP per mouse was injected with microinjuection syringe pump. **b,** The EGFP fluorescence was observed at ipsilateral L3 SchG 6-7 days after injection through fluorescent stereo microscope, showing the innervation to the hind paw. Scar bar : 200 μm. n = 2 mice.

Section 15. Immunostaining of TH

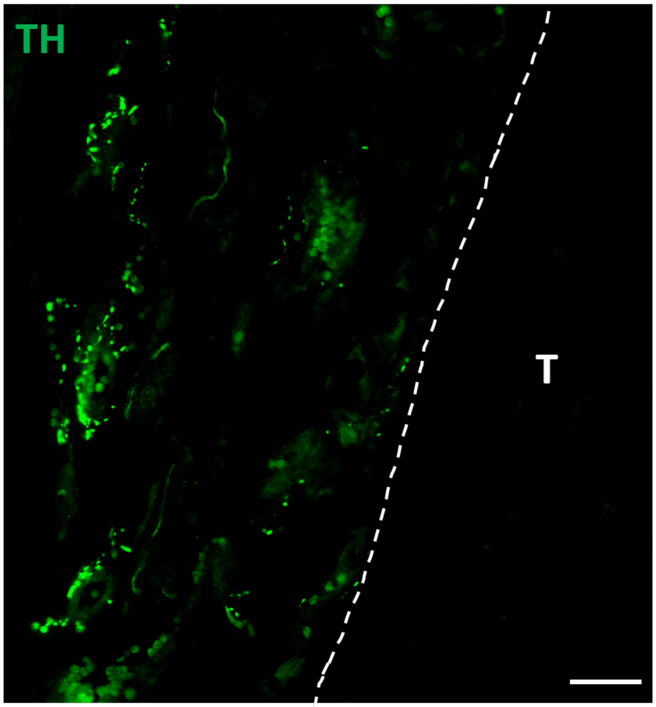

Figure S15 | The immunostaining of TH. The immunostaining verified that sympathetic nerve exists around floxer tendon which is the basis of ES on SchGs[3]. The right of dotted line was tendon. T means tendon. Scale bar: 25 μm. n = 2 mice.

Section 16. Electrode implantation

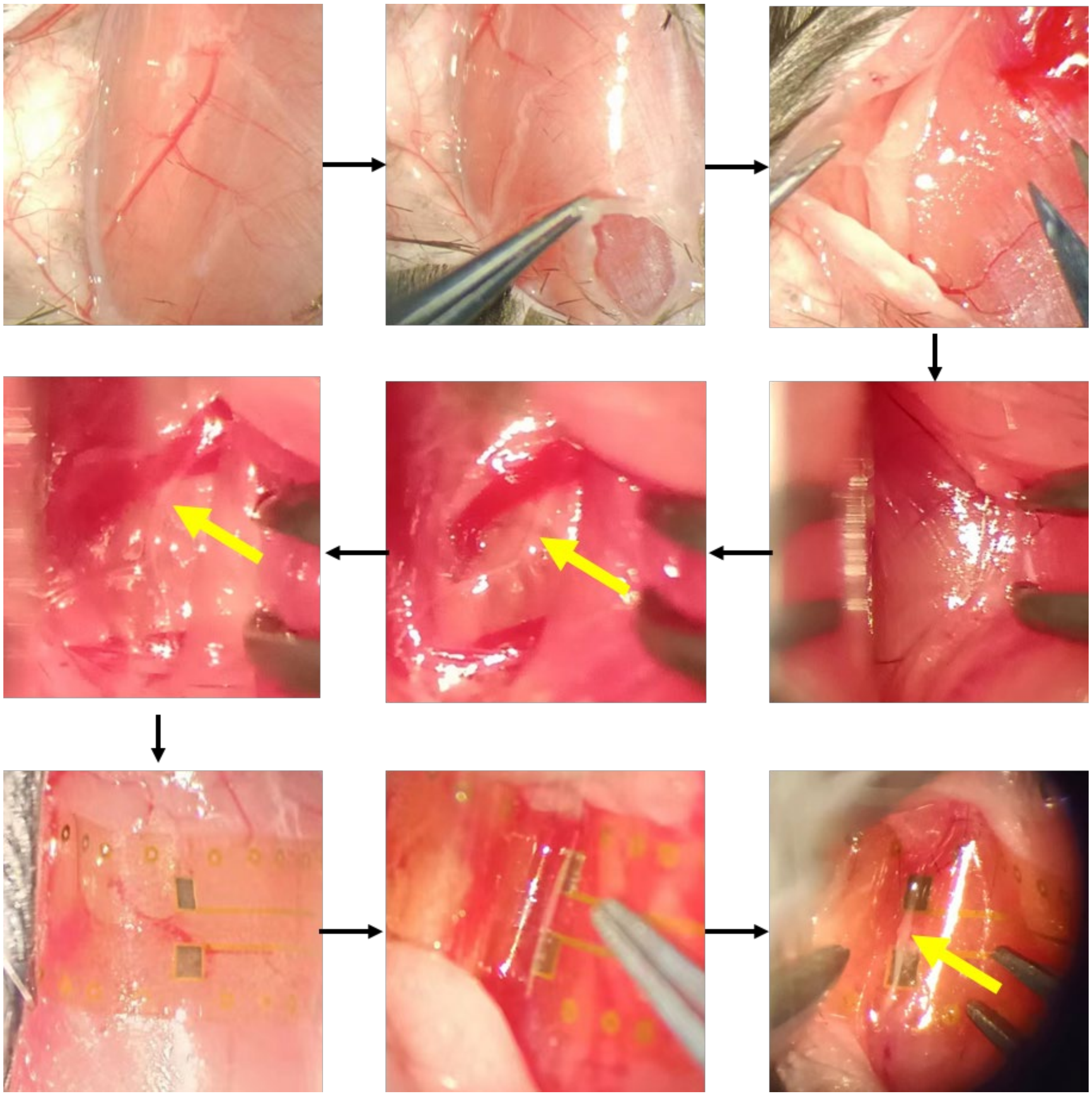

Figure S16 | The procedure of electrode implantation. The black arrows showed the order and the yellow arrows were sympathetic nerve. mice were fixed at a position of lateral decubitus. A 1-centimeter incision was made from the side of the mice to further separate the muscles and tissues. The sympathetic trunk running between the diaphragm and psoas major muscle was exposed behind the abdominal aorta. Then the right trunk was freed and flexible electrode was wrapped in specific segment.

Section 17. The influence of implantation

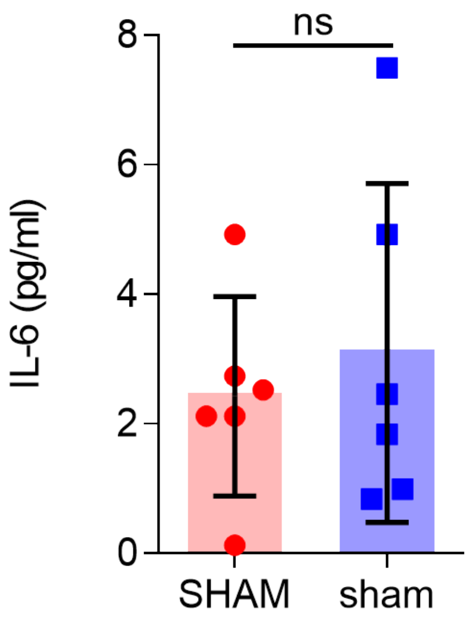

Figure S17 | The level of IL-6 in blood before and after implantation. The result between SHAM and sham groups showed that implantation had a negligible effect on the elevation of inflammatory cytokines. n = 6 mice; two-sided Student's unpaired t-test; ns, *p* = 0.6013. All data were expressed as the mean ± standard deviation (SD). ns, no significance.

Section 18. Low stimulation current caused little nerve damage

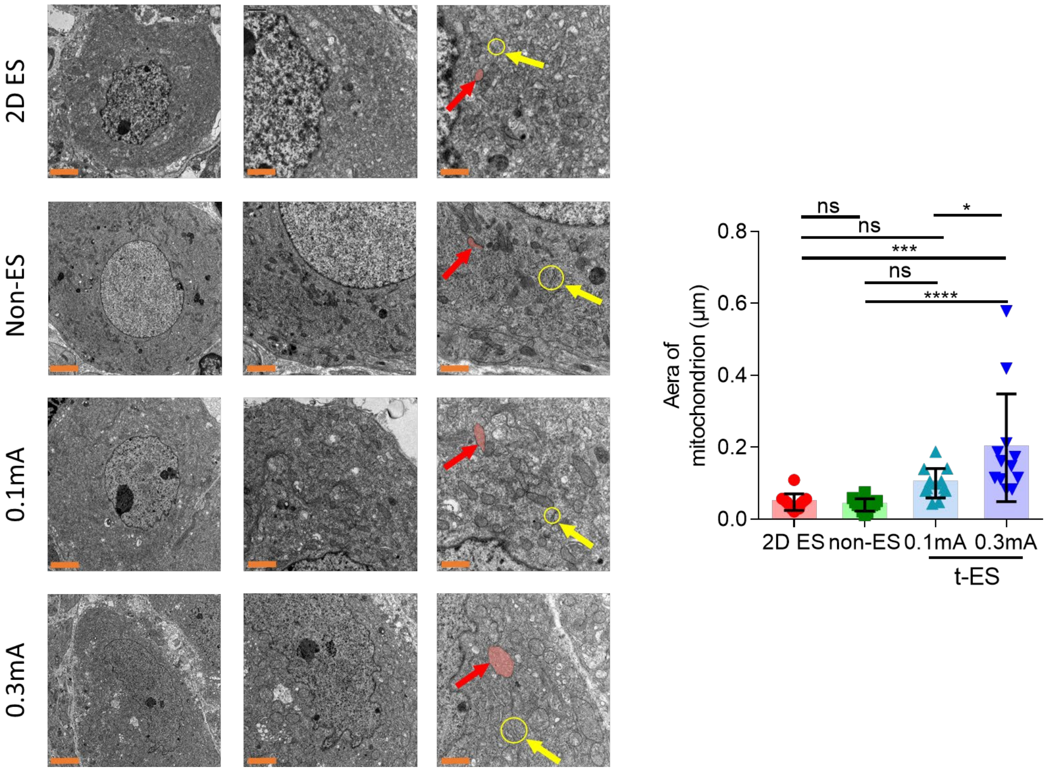

Figure S18 | Low stimulation current caused little damage on sympathetic neuron. Swelling rough endoplasmic reticulum and low electron density of mitochondrion in the 0.3 mA ES indicated that high current ES is damaging to sympathetic neurons. In contrast, the 0.1 mA ES and 2D ES caused little damage to neurons, like that of non-ES neurons. This suggests that 2D ES with lower stimulation current is expected to reduce nerve damage. Yellow arrow showed rough endoplasmic reticulum and red arrow with pink shadows showed mitochondrion. Aera of mitochondrion was also counted. The result showed that mitochondria in t-ES of 0.3 mA was expanded compared with 2D ES and t-ES of 0.1 mA, which implied that a higher current could cause damage to sympathetic neurons. n= 12 mitochondrions; one-way ANOVA; $F_{3,44} = 10.35$, $p < 0.0001$. post hoc Tukey test: ns, no significance, $p = 0.9955$ (2D ES vs. non-ES), $p = 0.3663$ (2D ES vs. 0.1mA t-ES), $p = 0.2534$ (non-ES vs. 0.1mA t-ES); *$p = 0.0189$; ***$P = 0.0001$; ****$p < 0.0001$. Scale bar from left to right: 2, 1, 0.5 μm (orange lines).

Section 19. Comprehensive assessment of damage after ES

We have demonstrated t-ES damage to SChG cells by HE staining and TEM. To thoroughly characterize and identify extent and nature of the damage on the SChG after ES. We have supplemented more in vitro and in vivo experiments to further characterize the damage of t-ES (**Figure S19-21**). Cell apoptosis occurs with tissue injuries. Cleaved caspase-3 (CC3) staining is the common marker of cell apoptosis, and we have added immunostaining of these on SChG to assess the damage of t-ES. Since PC-12 cells are widely used in neuroscience, including studies on neurotoxicity, neuroprotection and neurosecretion. CC3 staining and Calcein/PI test were also supplemented in the in vitro experiments to assess the damage extent of PC-12 cells with t-ES.

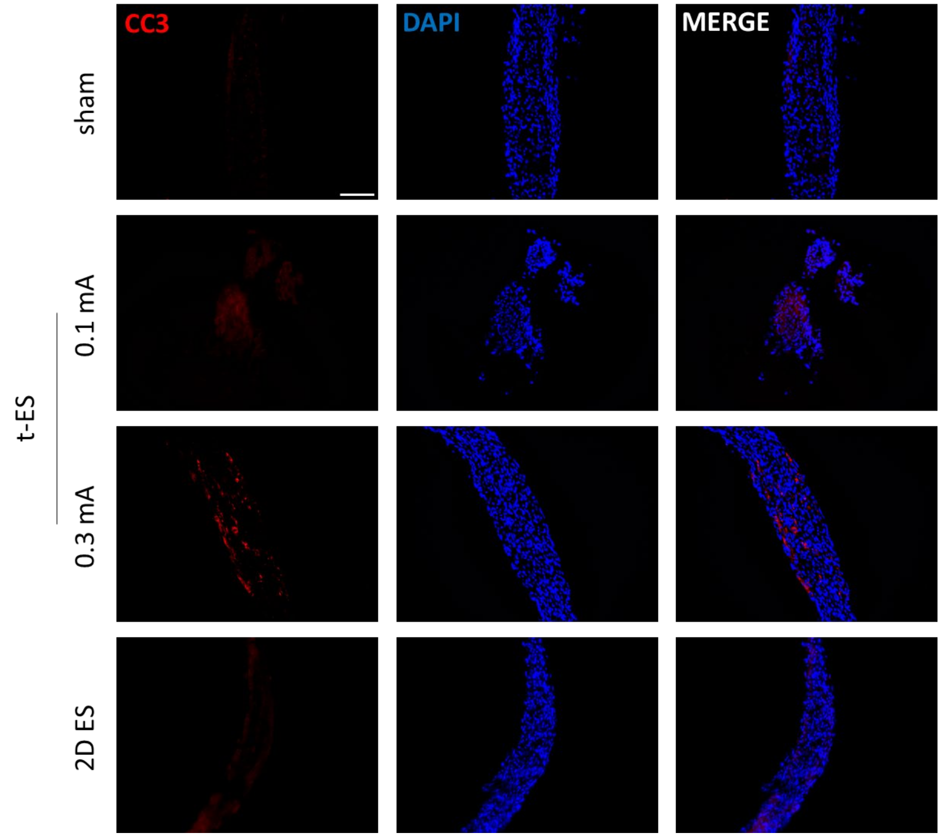

Figure S19 | The damage extent of neuron among the sham, t-ES (0.3, 0.1 mA) and 2D ES (~0.175 mA) groups. Cell apoptosis was observed after t-ES of 0.3 mA, while little apoptosis was observed in the other groups, suggesting that low currents are more suitable for biosafety. Scale bar: 50 μm. n = 2 mice.

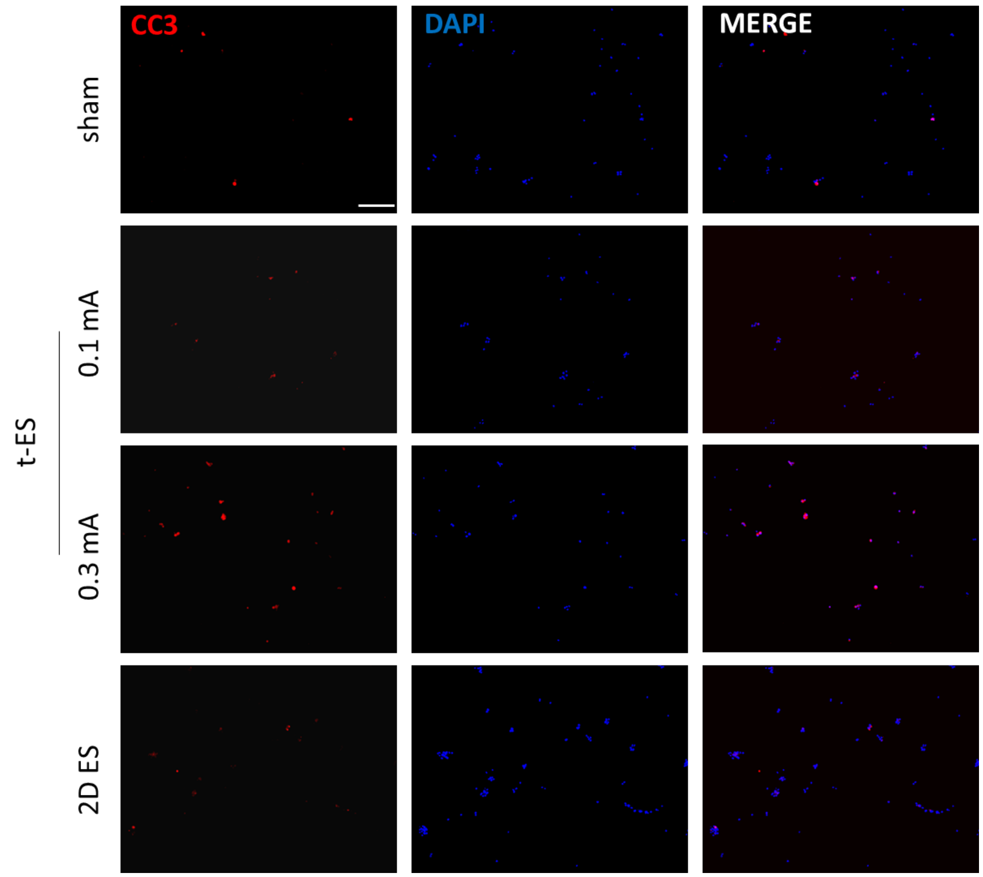

Figure S20 | The damage extent of PC-12 among the sham, t-ES (0.3, 0.1 mA) and 2D ES (~0.175 mA) groups. CC3 staining of PC-12 cells. CC3 was expressed obviously in t-ES of 0.3 mA, while it was little in other groups, which means that low stimulation currents show better biosafety. Scale bar: 50 μm. n = 6 biologically independent experiments.

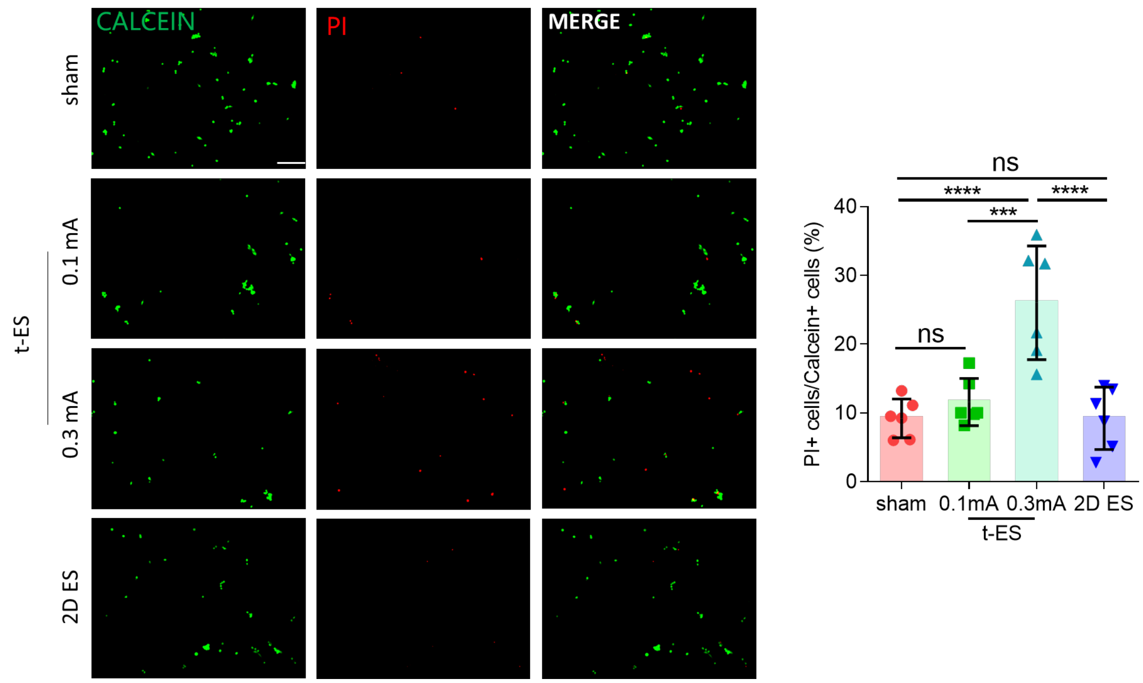

Figure S21 | The damage extent of PC-12 among the sham, t-ES (0.3, 0.1 mA) and 2D ES (~0.175 mA) groups. Calcein/PI test of PC-12 cells. Calcein represents live cells and PI represents dead cells. PI^{+} cells was observed increasing in t-ES of 0.3 mA compared with the other groups, which also showed that higher current caused more damage to neurons. Scale bar: 50 µm. The percentage of PI^{+} cells/$Calcein^{+}$ cells was observed increasing in t-ES of 0.3 mA compared with other groups, which indicated that higher current caused more damage to neurons. n=6 biologically independent experiments; One-way ANOVA; $F_{3,20}$= 14.45, p < 0.0001; post hoc Tukey test: ns, p = 0.8569 (sham vs. 0.1mA), p > 0.9999(sham vs. 2D ES); ***p =0.0006; ****p < 0.0001. Scale bar: 50 µm.

Section 20. Stimulation current threshold

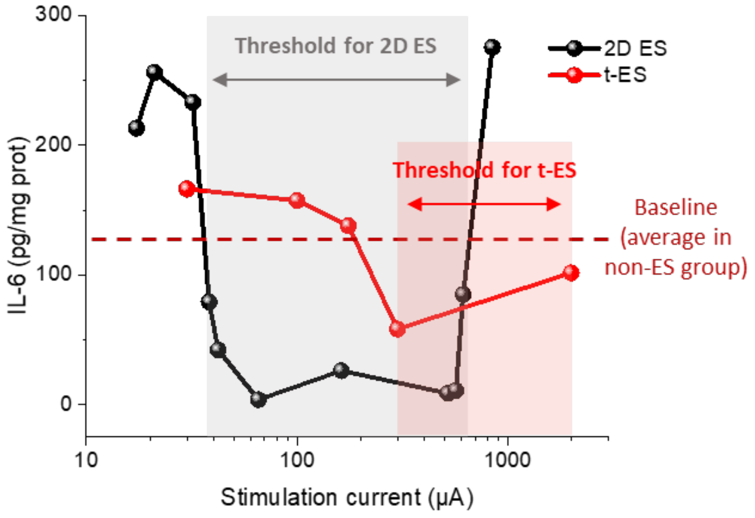

Figure S22 | Threshold range of stimulation current for both t-ES and 2D ES. The IL-6 concentrations under the effect of different stimulation current amplitudes were counted (same stimulation frequency and duration of 10 Hz, 15 min), and the results showed that the 2D ES threshold for effective inflammation inhibition ranged from 40~600 μA (average amplitude of ~175 μA), while t-ES for effective inflammation inhibition ranged from 300~2000 μA. Inflammatory cytokines appear elevated in response to smaller and larger current amplitudes (baselines for IL-6 is ~122 pg/mg prot).

Section 21. IL-6$^+$ and CD68$^+$ cell concentrations

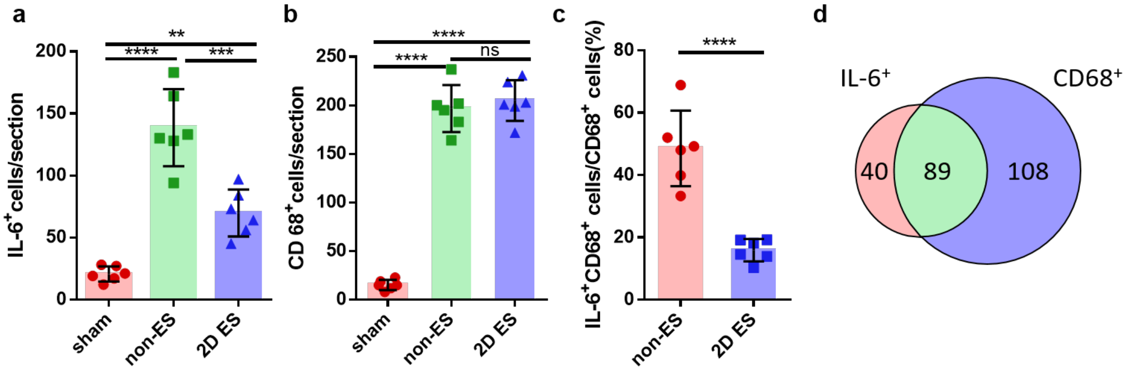

Figure S23 | Number of IL-6$^+$ and CD68$^+$ cells per section and percentage of IL-6$^+$CD68$^+$ cells in CD68$^+$ cells among the sham, 2D ES and non-ES groups. IL-6 was decreased obviously after 2D ES (**a**) and the number of CD68$^+$ cells has no change (**b**). **c,** The percentage of IL-6$^+$CD68$^+$ in the CD68$^+$cells. IL-6$^+$CD68$^+$ cells were decreased after 2D ES. **d,** The average number of IL-6$^+$CD68$^+$ cells in the IL-6$^+$ and CD68$^+$ cells in non-ES group. IL-6 were secreted mainly by macrophages. n = 6 mice; One-way ANOVA, for fig. a, $F_{2,15} = 46.50$, $p < 0.0001$; post hoc Tukey test: **$p = 0.0031$, ***$p = 0.0001$, ****$p < 0.0001$; for fig. b, $F_{2,15} = 197.4$, $p < 0.0001$; post hoc Tukey test: ****$p < 0.0001$; ns, $p = 0.7343$.Two-sided Student's unpaired t-test, for fig. c, ****$p < 0.0001$.

Section 22. Comprehensive analysis of inflammatory cytokines

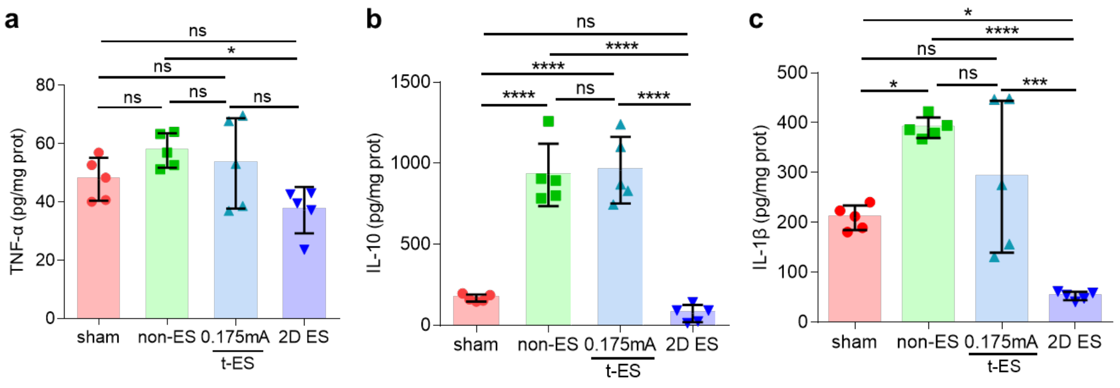

Figure S24 | The level of TNF-α, IL-10, IL-1β between sham, non-ES, t-ES (0.175 mA) and 2D ES (~0.175 mA) groups. a, The level of TNF-α had no significance between sham and non-ES groups, while 2D ES still decreased it compared with the non-ES group. **b-c,** 2D ES declined the level of IL-10, IL-1β compared with non-ES and 0.175 mA t-ES. n=5 mice; One-way ANOVA; for TNF-α, $F_{3,16}$ = 3.979, *p* = 0.027; post hoc Tukey test: **p* = 0.0224, ns, *p* = 0.4154 (sham vs. non-ES), *p* = 0.8182 (sham vs. 0.175mA), *p* = 0.3617 (sham vs. 2D ES), *p* =0.8922 (non-ES vs. 0.175mA), *p* = 0.0877 (0.175mA vs. 2D ES); for IL-10, $F_{3,16}$ = 54.82, $p < 0.0001$; post hoc Tukey test: ****$p < 0.0001$, ns, *p* = 0.7188 (sham vs. 2D ES), *p* = 0.9883 (non-ES vs. 0.175mA); for IL-1β, $F_{3,16}$ = 16.77, $p < 0.0001$; post hoc Tukey test: **p* = 0.0102 (sham vs. non-ES), **p* = 0.0268 (sham vs. 2D ES), ***p* = 0.0009, ****$p < 0.0001$, ns, *p* = 0.3708 (sham vs. 0.175mA), *p* = 0.2317 (non-ES vs. 0.175mA).

Section 23. Determine the effects of 2D ES on blood flow

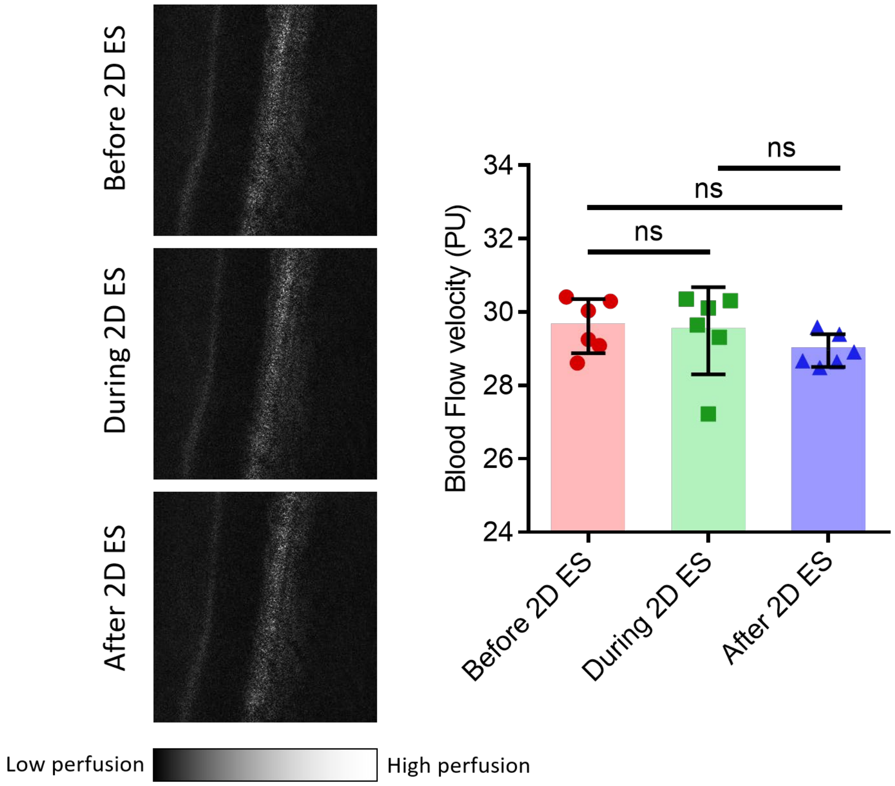

Figure S25 | 2D ES had no influence on blood flow of hind paws. Laser speckle was performed and illuminated that blood flow velocity had no significant change before, during and after 2D ES with the current allowed by the threshold range. n = 6 mice ; One-way ANOVA; $F_{2,15}$ =1.05, p = 0.3741; post hoc Tukey test: ns, p = 0.9645 (Before 2D ES vs. During 2D ES), p = 0.3839 (Before 2D ES vs. After 2D ES), p = 0.5243 (During 2D ES vs. After 2D ES).

Section 24. KEGG and GO analysis

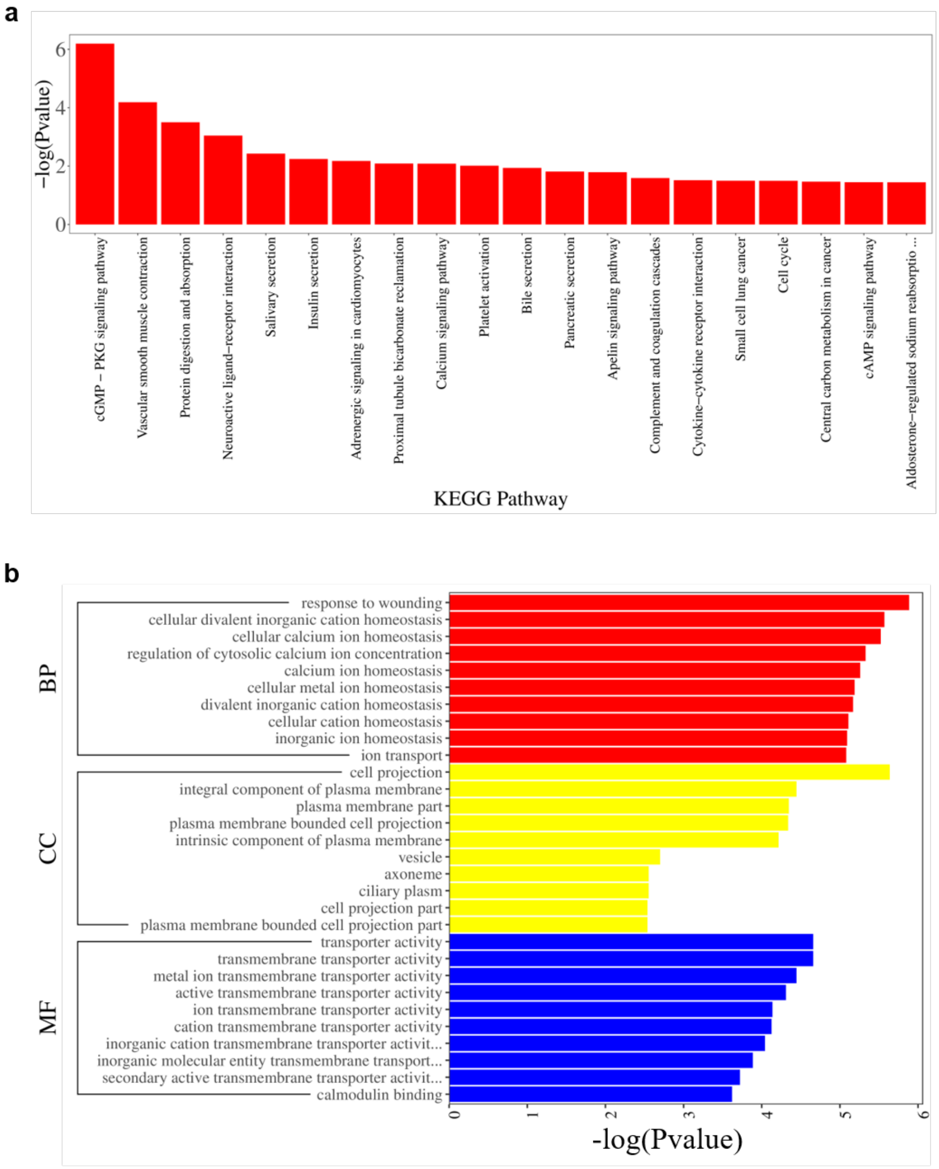

Figure S26 | Kyoto Encyclopedia of Genes and Genomes (KEGG) pathway analysis and Gene Ontology (GO) analysis. a, cAMP pathway enrichment results from KEGG pathway analysis[4]. **b,** Functional annotation of GO analysis showed the enrichment of DEGs from three aspects, biological process (BP) in red, cellular component (CC) in yellow and molecular function (MF) in blue.

Section 25. ADRB2$^+$CD68$^+$ cell concentration

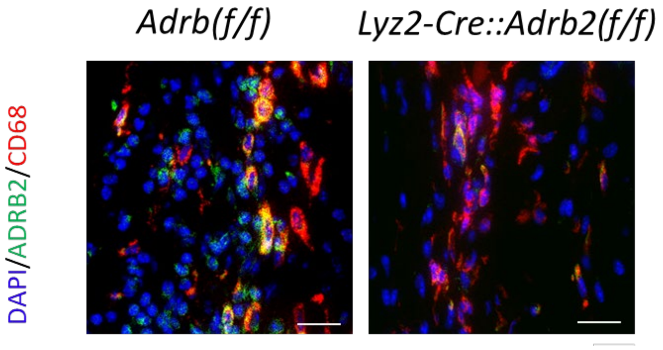

Figure S27 | The percentage of ADRB2$^+$CD68$^+$ cells in CD68$^+$ cells. The immunostaining including DAPI, ADRB2 and CD68. Scale bar: 100 μm. n = 6 mice.

Section 26. The effect of antagonists against ADRB2

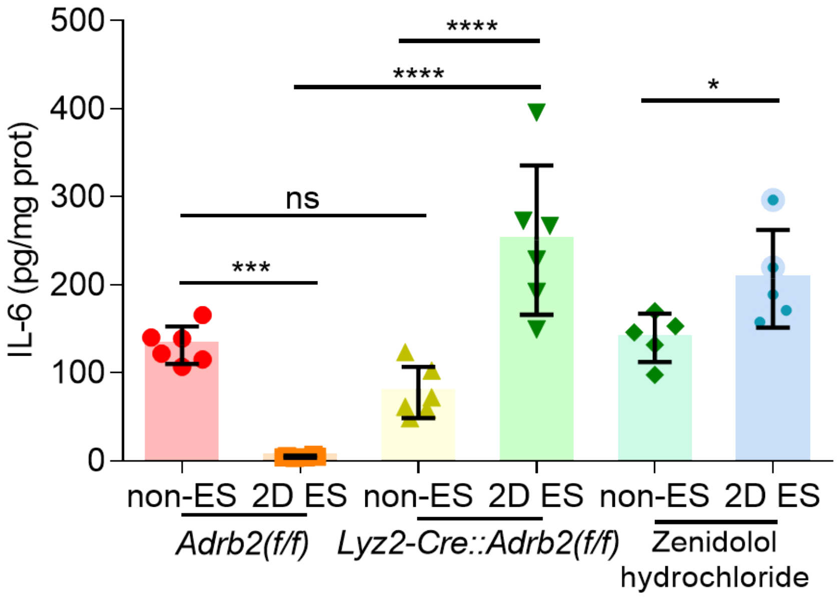

Figure S28 | The level change of IL-6 under the intervene of adrenoceptor. ELISA for IL-6 in the non-ES and 2D ES groups of *Lyz2-Cre::Adrb2(f/f)* and *Adrb2(f/f)* mice. The decrease of IL-6 was prevented after 2D ES in *Lyz2-Cre::Adrb2(f/f)* mice. 2D ES also failed to decreased the level of IL-6 after the ablation of ADRB2 through Zenidolol hydrochloride. Instead, it was increased compared with non-ES group. n=6 mice except for Zenidolol hydrochloride groups which of n was 5 mice. Two-way ANOVA; $F_{1,20} = 63.53$, $p < 0.0001$; post hoc Tukey test: ***$p < 0.001$; ****$p < 0.0001$; ns, $p = 0.2134$. For Zenidolol hydrochloride groups, two-sided Student's unpaired t-test, $p = 0.0413$.

Section 27. Benchmarking for electrostimulator systems

Table S1 provides statistics and comparisons of performance metrics for reported electrostimulator systems (including commercial stimulator, such as Intan RHS2000)[5-7]. The results showed that previous ES systems lacked specificity and did not act directly on the target nerve, while we isolated the sympathetic chain and wrapped it with flexible electrodes for specific stimulation. In addition, reported and commercial stimulators generally provide fixed rectangular charge-balanced biphasic pulse stimulation with low similarity to bioelectrical signals[5-7]. The non-specific of electroacupuncture and the low bionic similarity of stimulation spikes make them necessary to compensate with damagingly high amplitudes to achieve target symptom regulation. The proposed 2D neuromorphic electrostimulator is capable of providing programmable bionic spikes. Thanks to the nerve-specific stimulation and bionic spikes, 2D neuromorphic stimulator is able to inflammation inhibition with a record-low current (average of ~0.175 mA), which is expected to reduce nerve damage and subsequent complications. Furthermore, with the support of peripheral adapter boards and analyzers, 2D neuromorphic electrostimulator system has the ability to monitor loop currents in real time and provides timely feedback on the target nerve.

Electrostimulator system	Microcuff electrode	Electroacupuncture	Electroacupuncture	Commercial stimulators	2D Neuromorphic Electrostimulator
**Targeted symptom (diseases)**	Autoimmune diabetes	Systemic inflammation	Systemic inflammation	Inflammation in tendon injuries	Inflammation in tendon injuries
**Stimulus site**	Near pancreatic nerve	Acupoints (ST36, ST25)	Acupoints (ST36, ST25)	Sympathetic chain	**Sympathetic chain**
**Direct nerve stimulation**	Yes	No	No	Yes	**Yes**
**Stimulus signal**	Fixed rectangular charged-balanced biphasic pulses	Fixed rectangular charged-balanced biphasic pulses	Fixed rectangular charged-balanced biphasic pulses	Fixed rectangular charged-balanced biphasic pulses	**Programmable bionic spikes**
**Current amplitude**	0.45 mA	0.5-3 mA	0.5 mA	0.3-3 mA	**average of ~0.175 mA (0.04-0.6 mA)**
**Real-time monitoring of nerve stimulation**	incapable	incapable	incapable	incapable	**capable**
**References**	Nat. Biotechnol. 2019 [5]	Neuron 2020 [6]	Nature 2021 [7]	Our work	Our work

Table S1 | Performance benchmarks for electrostimulator systems.

Supplementary References

1 Zhang, E. *et al.* Tunable charge-trap memory based on few-layer MoS2. *ACS nano* **9**, 612-619 (2015).

2 Migliato Marega, G. *et al.* Logic-in-memory based on an atomically thin semiconductor. *Nature* **587**, 72-77 (2020).

3 Orimo, S. *et al.* Axonal α-synuclein aggregates herald centripetal degeneration of cardiac sympathetic nerve in Parkinson's disease. *Brain* **131**, 642-650 (2008).

4 Liu, X. *et al.* PDGF-loaded microneedles promote tendon healing through p38/cyclin D1 pathway mediated angiogenesis. *Materials Today Bio* **16**, 100428 (2022).

5 Guyot, M. *et al.* Pancreatic nerve electrostimulation inhibits recent-onset autoimmune diabetes. *Nat. Biotechnol.* **37**, 1446-1451 (2019).

6 Liu, S. *et al.* Somatotopic organization and intensity dependence in driving distinct NPY-expressing sympathetic pathways by electroacupuncture. *Neuron* **108**, 436-450. e437 (2020).

7 Liu, S. *et al.* A neuroanatomical basis for electroacupuncture to drive the vagal–adrenal axis. *Nature* **598**, 641-645 (2021).
