# Supplementary material for: Neuromorphic electro-stimulation based on atomically thin semiconductor for damage-free inflammation inhibition
Source: Nat Commun. 2024 Feb 13;15:1327. doi: 10.1038/s41467-024-45590-8 (PMC10864345; doi:10.1038/s41467-024-45590-8)
Supplement: Supplementary file 3 — Reporting Summary [file 41467_2024_45590_MOESM3_ESM.pdf]

Corresponding author(s): Shuiyuan Wang, Peng Zhou

Last updated by author(s): Jan 14, 2024

# Reporting Summary

Nature Portfolio wishes to improve the reproducibility of the work that we publish. This form provides structure for consistency and transparency in reporting. For further information on Nature Portfolio policies, see our Editorial Policies and the Editorial Policy Checklist.

## Statistics

For all statistical analyses, confirm that the following items are present in the figure legend, table legend, main text, or Methods section.

| n/a | Confirmed | |
|---|---|---|
| ☐ | ☒ | The exact sample size (*n*) for each experimental group/condition, given as a discrete number and unit of measurement |
| ☐ | ☒ | A statement on whether measurements were taken from distinct samples or whether the same sample was measured repeatedly |
| ☐ | ☒ | The statistical test(s) used AND whether they are one- or two-sided<br>*Only common tests should be described solely by name; describe more complex techniques in the Methods section.* |
| ☐ | ☒ | A description of all covariates tested |
| ☐ | ☒ | A description of any assumptions or corrections, such as tests of normality and adjustment for multiple comparisons |
| ☐ | ☒ | A full description of the statistical parameters including central tendency (e.g. means) or other basic estimates (e.g. regression coefficient) AND variation (e.g. standard deviation) or associated estimates of uncertainty (e.g. confidence intervals) |
| ☒ | ☐ | For null hypothesis testing, the test statistic (e.g. *F*, *t*, *r*) with confidence intervals, effect sizes, degrees of freedom and *P* value noted<br>*Give P values as exact values whenever suitable.* |
| ☒ | ☐ | For Bayesian analysis, information on the choice of priors and Markov chain Monte Carlo settings |
| ☐ | ☒ | For hierarchical and complex designs, identification of the appropriate level for tests and full reporting of outcomes |
| ☒ | ☐ | Estimates of effect sizes (e.g. Cohen's *d*, Pearson's *r*), indicating how they were calculated |

*Our web collection on statistics for biologists contains articles on many of the points above.*

## Software and code

Policy information about availability of computer code

| | |
|---|---|
| Data collection | no software are used |
| Data analysis | GraphPad Prism 9.4.1 |

For manuscripts utilizing custom algorithms or software that are central to the research but not yet described in published literature, software must be made available to editors and reviewers. We strongly encourage code deposition in a community repository (e.g. GitHub). See the Nature Portfolio guidelines for submitting code & software for further information.

## Data

Policy information about availability of data

All manuscripts must include a data availability statement. This statement should provide the following information, where applicable:

- Accession codes, unique identifiers, or web links for publicly available datasets
- A description of any restrictions on data availability
- For clinical datasets or third party data, please ensure that the statement adheres to our policy

The data that support the findings of this study are available from the corresponding authors upon reasonable request.

## Research involving human participants, their data, or biological material

Policy information about studies with human participants or human data. See also policy information about sex, gender (identity/presentation), and sexual orientation and race, ethnicity and racism.

| | |
|---|---|
| Reporting on sex and gender | N.A. |
| Reporting on race, ethnicity, or other socially relevant groupings | N.A |
| Population characteristics | N. A. |
| Recruitment | N. A. |
| Ethics oversight | N. A. |

Note that full information on the approval of the study protocol must also be provided in the manuscript.

# Field-specific reporting

Please select the one below that is the best fit for your research. If you are not sure, read the appropriate sections before making your selection.

☒ Life sciences ☐ Behavioural & social sciences ☐ Ecological, evolutionary & environmental sciences

For a reference copy of the document with all sections, see nature.com/documents/nr-reporting-summary-flat.pdf

# Life sciences study design

All studies must disclose on these points even when the disclosure is negative.

| | |
|---|---|
| Sample size | Each sample size was chose at 6 just as previous study on electrostimulation and tendon injury. |
| Data exclusions | No data was excluded. |
| Replication | Experimental findings were replicable. |
| Randomization | The experimental animals were divided by a random chart method. |
| Blinding | The investigators were blinded to group allocation during data collection and/or analysis. |

# Reporting for specific materials, systems and methods

We require information from authors about some types of materials, experimental systems and methods used in many studies. Here, indicate whether each material, system or method listed is relevant to your study. If you are not sure if a list item applies to your research, read the appropriate section before selecting a response.

## Materials & experimental systems

| n/a | Involved in the study | |
|---|---|---|
| ☐ | ☒ | Antibodies |
| ☐ | ☒ | Eukaryotic cell lines |
| ☒ | ☐ | Palaeontology and archaeology |
| ☐ | ☒ | Animals and other organisms |
| ☒ | ☐ | Clinical data |
| ☒ | ☐ | Dual use research of concern |
| ☒ | ☐ | Plants |

## Methods

| n/a | Involved in the study | |
|---|---|---|
| ☒ | ☐ | ChIP-seq |
| ☒ | ☐ | Flow cytometry |
| ☒ | ☐ | MRI-based neuroimaging |

## Antibodies

| | |
|---|---|
| Antibodies used | The primary antibodies used in this study included the following: rat anti-CD68 (NBP2-33337, Novus Biologicals), rabbit anti-IL-6 (ab290735, Abcam), rabbit anti-ADRB2 (ab182136, Abcam), rabbit anti-Cleaved Caspase-3 (Cat. # 9661S, CST). Secondary antibodies conjugated to Alexa Fluor 488 (AF488), and AF594 included the following: AF488 donkey anti-rat (34406ES60, YEASEN) and AF594 donkey anti-rabbit (A21207, Invitrogen) |

| | |
|---|---|
| Validation | Rat anti-CD68 (NBP2-33337, Novus Biologicals), rabbit anti-IL-6 (ab290735, Abcam), rabbit anti-ADRB2 (ab182136, Abcam) and rabbit anti-Cleaved Caspase-3 (Cat. # 9661S, CST) were used in the study which were all valiated in the manufacturer's website. |

## Eukaryotic cell lines

Policy information about cell lines and Sex and Gender in Research

| | |
|---|---|
| Cell line source(s) | The cell line of highly differentiated PC-12 cellscomes from a transplantable male rat adrenal pheochromocytoma. |
| Authentication | The cell line was authenticated by the National Collection of Authenticated Cell Cultures. |
| Mycoplasma contamination | The cell line was tested negative for mycoplasma contamination. |
| Commonly misidentified lines (See ICLAC register) | N.A. |

## Animals and other research organisms

Policy information about studies involving animals; ARRIVE guidelines recommended for reporting animal research, and Sex and Gender in Research

| | |
|---|---|
| Laboratory animals | Lyz2-Cre mice (B6.129P2-Lyz2tm1(cre)Ifo/J, stock no: 004781) were purchased from The Jackson Laboratory. Adrb2(fl/fl) (B6/JGpt-Adrb2em1Cflox/Gpt, stock no: T052308) and C57BL/6J (wild type, WT) mice were purchased from GemPharmatech.Mice of both sexes were utilized for the experiments unless specified. The age of the animals were 8-10 weeks. |
| Wild animals | The study did not involve wild animals. |
| Reporting on sex | This information has not been collected. |
| Field-collected samples | The study did not involve samples collected from the field. |
| Ethics oversight | All mouse experiments were conducted in accordance with the guidelines of the Ethics Committee of Shanghai Sixth People's Hospital. The study was approved by the Ethics Committee of Shanghai Sixth People's Hospital (Reference Number: 2022-0688). |

Note that full information on the approval of the study protocol must also be provided in the manuscript.

## Plants

| | |
|---|---|
| Seed stocks | N. A. |
| Novel plant genotypes | N. A. |
| Authentication | N. A. |
